# Can positive affect attenuate (persistent) pain? State of the art and clinical implications

**DOI:** 10.1007/s11926-017-0703-3

**Published:** 2017-11-09

**Authors:** Marjolein M. Hanssen, Madelon L. Peters, Jantine J. Boselie, Ann Meulders

**Affiliations:** 1 Clinical Psychological Science, Research Group Behavioral Medicine, Maastricht University, P.O. 616, 6200 MD Maastricht, The Netherlands; 2 Research Group Health Psychology, KU Leuven, Leuven, Belgium

**Keywords:** Positive affect, Positive psychology interventions, Persistent pain, Resilience, Pain

## Abstract

**Purpose of Review:**

Pain is an intense experience that can place a heavy burden on peoples’ lives. The identification of psychosocial risk factors led to the development of effective pain treatments. However, effect sizes are modest. Accumulating evidence suggests that enhancing protective factors might also impact on (well-being despite) pain. Recent findings on positive affect (interventions) towards pain-related outcomes will be reviewed, and new avenues for treatment of persistent pain will be discussed.

**Recent Findings:**

Positive affect significantly attenuates the experience of pain in healthy and clinical populations. Positive affect interventions effectively reduce pain sensitivity and bolster well-being despite pain. Through both psychological and (neuro-)biological pathways, but also through its effect on central treatment processes such as inhibitory learning, positive affect can optimize the efficacy of existing treatments.

**Summary:**

Comprehensive understanding of the unique roles and dynamic interplay of positive and negative affect in moderating pain may optimize the treatment of (persistent) pain.

## Introduction

Pain is a complex experience that may lead to various limitations in daily life [1]. According to the International Association for the Study of Pain [2], pain is “an unpleasant sensory and emotional experience associated with actual or potential tissue damage.” This definition emphasizes that pain is in essence both a sensory and an emotional experience for which tissue damage is not a prerequisite. Several decades of pain research support a biopsychosocial conceptualization in which pain is a result of the interplay between biological, psychological, and social factors [3–5]. For instance, it has been shown that pain-related fear can be even more disabling than pain itself [6]. The identification of psychological factors that negatively influence the experience of pain led to the development of effective psychological pain treatments aimed at reducing overly negative cognitions/emotions and maladaptive behaviors. However, a subset of patients does not complete such a treatment or fails to achieve satisfactory results during or after the treatment [7–9]. Thus, there is considerable room for improvement in psychological pain treatment targeting risk factors for increased suffering due to persistent pain.

In pain psychology, as in other areas of clinical-psychological and psychiatric research, the exclusive focus on individual vulnerabilities (“fixing what’s wrong”) is recently being extended with an interest in what protects people in challenging circumstances (“building what’s strong”) [10, 11]. Resilient individuals, described as individuals who flexibly adapt to adverse circumstances (e.g., individuals who successfully deal with the limitations accompanying persistent pain) have become a source of inspiration in (pain) psychology [12, 13]. Sturgeon and Zautra [14] argued for systematic research of resources/mechanisms of both vulnerability and resilience to disentangle what leads to outcomes of recovery (return to homeostasis), sustainability (enduring engagement in valued activities), and growth (benefit finding/post-traumatic growth) in the field of pain. Evidence for positive affect as a resilience factor in pain has accumulated [15••, 16••]. Moreover, interventions based on positive psychology, or the study of positive human functioning [17], can diminish pain intensity and promote positive affect and well-being despite pain [18•, 19]. In what follows, we will review the recent developments after a brief conceptualization of the construct “affect” is given.

## Positive Affect: a Conceptualization

The term “affect” refers to both short-lasting emotions as well as more stable moods. While an emotion is an affective state that has a clear focus (response to specific events), moods are more diffuse [20, 21].

Affective states are considered to vary along several dimensions [20, 22]. First, the dimension of *valence* indicates the subjective evaluation of an experienced state. While positive affect reflects pleasant feelings (e.g., joy, amusement), negative affect reflects unpleasant feelings (e.g., fear, anger) [23]. Second, the dimension of *arousal* refers to the activation of the sympathetic nervous system in response to an event, generally experienced as (high/low) levels of energy. In response to threat, increased activity in the sympathetic nervous system triggers physiological hyperarousal, also known as the fight-flight response [24]. The level of arousal is immediately linked to motivation. Third, the motivational component of emotions refers to the *action tendencies* that accompany emotions. Today, it is widely accepted that action tendencies, such as escape in response to fear or approach behavior in response to enthusiasm, is what makes emotions evolutionary adaptive [20–22]. The adaptive nature of emotions materializes in the way they control attention, thoughts, and behavior. For instance, emotions in response to pain guide pain-related thoughts, interrupt ongoing behavior, and disengage attention from stimuli other than pain. In case of acute pain, this narrowing effect of negative emotions has a protective function. When affective states are very intense or prolonged, however, they might exceed their evolutionary function of promoting adaptation. This undesirable side effect is often observed in patients with persistent pain in which adaptation is compromised as a result of misdirected attempts to control or avoid pain [25•, 26].

A substantial part of the literature on affect has been dedicated to clarifying the structure of the construct. Both bipolar and bifactorial models have been proposed as a framework for positive and negative affect. On the one hand, it was found that decreases in negative affective states associated with pain coincide with increased positive affect, hereby supporting the bipolar conceptualization [27]. On the other hand, evidence also exists supporting the bifactorial model, that is, certain positive (or negative) events seem to bring about changes in positive (or negative) affect only [28]. In a recent review on positive affect and pain, Finan and Garland [16••] concluded that positive affect and negative affect are only moderately correlated both in healthy participants following experimental pain induction and in chronic pain patients. According to the Dynamic Model of Affect [29, 30], both conceptualizations are useful in describing daily fluctuations in affect, depending on the circumstances. While positive affect and negative affect tend to show modest correlations under normal circumstances, they may be highly correlated in adverse situations (e.g., a pain flare up). In this view, resilience may reflect the ability to maintain high levels of positive affect despite elevations in negative affect [30, 31]. Although positive affect and negative affect might show substantial overlap under certain conditions, they seem to be separable affective states that have unique roles in modulating the experience of pain.

## Positive Affect as a Resilience Factor for (Persistent) Pain

The role of negative emotions in the experience of pain is well-documented [32, 33]. Negative emotional responses or low mood states, such as anger or depression, can intensify the pain experience [34–36]. The role of pain-related fear in particular has been emphasized in acute [37, 38] as well as in chronic pain [26, 39]. Fear and anxiety—in case of mild levels and when limited in time—actually instigate recovery behaviors. However, when intense or prolonged in time, these emotions instigate a downward spiral, which in turn may complicate the experience of persistent pain. More specifically, fear-avoidance patterns have been shown to predate future depressive feelings, pain disability, and disuse in the long run [25, 26]. For example, fear of falling correlates significantly with foot impairment, foot disability, and walking velocity in women with established rheumatoid arthritis [40].

Evidence for the role of positive affect in the experience of pain is accumulating. In contrast to the typical pain-enhancing role of negative affect, positive affect seems to be related to diminished pain [31, 41]. Moreover, positive affect enhances adaptation to persistent pain as exemplified by its negative associations with pain intensity, negative affect, sleep disturbances, and physical dysfunctions in pain patients [42–44]. Positive affect was also found to buffer the negative effects of pain on general functioning and well-being in patients with rheumatoid arthritis [31, 45] and other persistent pain conditions [31, 46].

Experimental work confirms the causal role of positive affect towards pain-related outcomes. Generally, it is found that successfully inducing positive affect using for instance affective slides/pictures/video fragments leads to a less intense pain experience than inducing negative or neutral affect in healthy participants undergoing an experimental pain task [47, 48]. In addition, experimentally inducing positive affect (and cognitions) by means of writing and visualizing about a future in which most life goals have been accomplished (Best Possible Self (BPS) exercise) led not only to reduced pain sensitivity [49] but also to less pain-induced cognitive interference in healthy participants [50, 51]. Particularly positive emotions with moderate to high levels of arousal (e.g., joy, happiness) seem to be able to produce this effect [52].

The BPS exercise is an example of a Positive Psychology Intervention (PPI), a psychological intervention (training/exercise/therapy) aimed at cultivating positive emotions, cognitions, and behavior [53]. These PPIs also proved to be associated with pain-related outcomes in (sub-)clinical populations. For instance, Hausmann and colleagues [19] performed secondary analyses on a subset of a community sample that participated in an online positive psychology program comprising several exercises (e.g., practicing gratitude or identifying strengths). The intervention led to increased positive mood and reduced bodily pain at the end of the 6-week interventions and at a 6-month follow-up in the individuals reporting current pain.

Another computer-based PPI also proved to be successful for individuals with physical disability and chronic pain [54]. Reduced levels of several pain outcomes (pain intensity, pain interference, and depressive feelings) were recorded post intervention and at a 2.5-month follow-up. Carson and colleagues [55] found that an 8-week program focusing on one specific exercise, “practicing kindness,” also led to improvements in pain and distress in chronic low back pain patients. In a large-scale randomized clinical trial (RCT), an 8-week online PPI “Happy despite Pain” consisting of four modules (self-compassion, “three good things,” savoring, and the BPS exercise) was compared to a risk-based cognitive-behavioral intervention in a sample predominantly consisting of fibromyalgia patients. Although no reduction in pain intensity was reported, both active interventions effectively reduced depression and increased happiness in contrast to individuals in the waiting list control group, who reported no significant changes [18•].

The benefits of inducing positive affect on pain-related outcomes were also confirmed in pain patients receiving PPIs using modern technology. For instance, a text message-based social support intervention succeeded in increasing positive affect and reducing perceptions of pain and pain interference in chronic non-cancer pain patients [56]. Additionally, Herrero and colleagues used virtual reality (VR) to induce positive affect in fibromyalgia patients. A predefined positive nature scenario was projected in the VR surrounding, including music with positive valence and high arousal. This VR intervention was successful in improving quality of life and reducing negative emotions as well as dysfunctional coping strategies [57]. Thus, ample evidence, also in (sub-)clinical populations, endorses the causal role of positive affect in reducing undesirable pain-related outcomes.

## Pathways of Positive Affect Towards Pain-Related Outcomes

### A (Neuro)Biological Pathway

Biological processes have been suggested to explain health-promoting effects of positive affect [58]. Nervous, endocrine, and immune processes play a role in the experience of (persistent) pain, which is typically intertwined with a complex stress response. Without providing an exhaustive description of biological processes in (persistent) pain, relevant connections with positive affect are highlighted below.

First, processing of nociceptive stimuli involves several key brain regions, including areas associated with emotional processes [59]. Persistent pain may be accompanied by structural alternation in (the connectivity of) key regions such as the prefrontal cortex or limbic structures [60, 61]. In addition, brain regions processing sensory, emotional, and cognitive information are involved in descending modulation of pain [62]. Positive affect has been related to both spinal and supraspinal pain modulation. For instance, inducing positive affect by means of emotional pictures was associated with a decrease in spinal nociceptive reflex, an index of descending pain modulation [63]. A study by Roy and colleagues showed that a positive affect induction modulates pain through multiple mechanisms, including supraspinal modulation [64].

Second, nociception is typically accompanied by a stress response (i.e., increase in cortisol), aimed at coping with the injury. Persistent exposure to stressors can lead to maladaptive neurobiological changes in pain processing pathways, in particular the descending inhibitory pain pathway, resulting in enhanced pain sensitivity (i.e., stress-induced hyperalgesia) [65]. Typically, an inverse relationship between positive affect and levels of cortisol has been found. This link seems to be independent from levels of negative affect [66].

Third, effects of pro-inflammatory cytokines are often observed in conditions of persistent pain such as osteoarthritis. Pro-inflammatory cytokines not only sensitize peripheral nerve fibers [67], but also lead to centrally maintained hyperalgesic states [68, 69]. It seems that positive affect may attenuate both peripheral and central pain facilitation through a reduction of inflammation [58]. Recent evidence however also shows that inflammation might modulate the mesolimbic reward system, hereby impacting on the responsiveness to pleasurable stimuli [70].

These findings thus suggest that positive affect may influence nervous, endocrine, and immune responses, as well as their complex interplay in relation to the experience of pain.

### A Pathway Through Countering Fear-Avoidance Patterns

Positive affect may prevent individuals from getting caught in a downward spiral that fuels negative pain-related outcomes. Based on the (partial) overlap with negative affect, positive affect might prevent that individuals adopt fear-avoidance patterns that promote maladjustment to pain. Additionally, the unique characteristics of positive affect may also fuel a positive upward spiral bolstering positive adaptation to persistent pain. More specifically, the broadening effect of positive emotions as described in the Broaden-and-Built theory can counter the narrowing effect of negative emotions [29, 71].

The Broaden-and-Built Model of Fredrickson is one of the most influential theories that have been introduced to explain the beneficial effects of positive emotions [72, 73]. Central to this model is the broadening effect of positive emotions on attention, thoughts, and behavior. In the long run, the broadening effect of positive emotions contributes to building enduring physical, cognitive, and social resources. The “undoing hypothesis” explains how long-term positive effects of positive emotions can serve as an antidote against the immediate effects of negative emotions [71]. Reduced positive emotions that might result from the experience of pain may increase the vulnerability to further negative emotions. Experimental and clinical work provides evidence for the protective role of positive affect in persistent pain based on its potential to broaden attention, cognition, and behavior.

First, experimental work shows that positive affect broadens the scope of (visual) *attention* [73, 74]. Attentional biases for negative stimuli or hypervigilance for pain are often found in patients with persistent pain [75]. Positive affect may counter the narrowing effect of fear on attention. For instance, positive affect may help patients with rheumatoid arthritis to not only be focused on pain and its negative consequences but also to be attentive for positive aspects of the situation and/or for non-pain-related stimuli, a pattern associated with better adjustment to pain [25, 26].

Second, positive emotions can buffer negative pain-related *cognitions*, such as pain catastrophizing. For instance, inducing positive emotions in healthy participants led to lower pain reports in an experimental pain task through diminished levels of pain catastrophizing [49]. The positive psychology intervention “Happy despite Pain” also significantly impacted on the level of pain catastrophizing in patients with persistent pain [18, 76]. Increases in positive emotions might particularly decrease catastrophic thoughts because they reduce ruminating and feeling helpless [15••]. In addition, positive emotions might promote positive and neutral (re-)appraisal processes related to pain. A positive (non-negative) appraisal style has been advanced as a key mechanism in resilience to adverse stimuli such as pain [11].

Third, positive affect might reduce engaging rigidly in specific action tendencies and create *behavioral flexibility* [21, 71]. While negative affect in response to pain leads to action tendencies primarily aimed at avoiding pain or reinjury, positive affect enhances (continued) engagement in valued activities despite the experience of pain. White and colleagues [77] observed significantly higher daily activity as measured by the number of steps in knee osteoarthritis patients with high positive affect as opposed to low positive affect. The number of steps in patients with high positive affect did not depend on their pain levels. Additionally, a VR study in fibromyalgia patients showed that inducing positive affect leads to enhanced activity engagement and motivation [57].

### A Pathway Through Improved Learning

The ability to accurately predict pain urges us to take action and protect ourselves from impending or actual bodily threat. Pavlovian (or classical) conditioning is the prime mechanism enabling us to predict (increases in) pain [78]. More specifically, an initially neutral stimulus (conditioned stimulus; CS) that has preceded or co-occurred with pain (unconditioned stimulus; US) may come to elicit fear and spur avoidance behavior (conditioned response; CR) [79]. Accurately identifying unique predictors of pain is challenging, because typically multiple environmental stimuli as well as interoceptive and proprioceptive events are present during a pain episode. Failure to identify the actual predictors of pain may lead to the spreading of fear and threat beliefs to a wide range of stimuli that were present during the pain experience, resulting in sustained anxiety [80, 81], and/or an increase in the frequency of fearful responding and persistent avoidance behavior. It is commonly accepted that threat expectancy learning (awareness of the CS-US contingency) is necessary to generate conditioned fear (i.e., arousal component of fear) and avoidance behavior (i.e., behavioral component of fear). Accumulating evidence shows that patients with persistent pain display impaired safety learning and excessive generalization [82–85]. There is preliminary experimental evidence suggesting that increasing positive affect may alter at least two learning processes: generalization and extinction (inhibitory) learning.

First, positive affect may limit *excessive generalization* of pain-related fear. Inducing positive affect in healthy individuals using the BPS exercise protected against generalization of fear to technically safe stimuli [86•]. More specifically, in a learning task, in which one movement was conditioned to elicit fear (by repeatedly pairing it with a painful electrocutaneous stimulus) and another movement was not. Novel movements (generalization stimuli, GSs) with varying levels of similarity with the original movements were subsequently tested. Results showed that positive affect especially decreased fear for those GS movements that were more similar to the original non-painful movement. Generalization is an adaptive process, enabling us to extrapolate the threat value from one situation to a similar one; excessive generalization to technically safe stimuli however may be maladaptive and lead to persistent avoidance behavior. Positive affect seems to leave the adaptive side of generalization intact, but eliminates maladaptive “overgeneralization.”

Second, it has been argued that positive affect might enhance a central treatment process, namely *inhibitory learning* [87••]. Pavlovian extinction learning is the laboratory analogue of clinical exposure treatment. Nowadays, extinction learning is not viewed as erasing the originally acquired association (e.g., a bending movement → pain; CS-US association), but the learning of a new association (e.g., a bending movement → no pain; CS-noUS association) that *inhibits* the behavioral expression of the first-learned association. Therefore, this type of learning is also referred to as inhibitory learning. Exposure therapy has a strong pedigree as one of the most effective strategies to reduce pain-related fear and associated disabling behaviors in highly fearful chronic pain patients [88]. In practice, patients are asked to perform feared activities (e.g., bending to pick something up) without the feared catastrophe occurring (e.g., the spine snapping). This disconfirmation experience allows the patient to learn that these activities are actually safe (i.e., safety learning). Gradually, this new learning will reduce catastrophic thoughts, pain-related fear, and functional disability [25], and although not an explicit goal before treatment, it often reduces pain as well. Experimental evidence is emerging to support the notion that positive affect may enhance inhibitory learning. For example, trait-positive affect has been shown to protect against deficient safety learning during extinction in healthy high-anxious individuals [89]. Moreover, experimental work also shows that positive affect might prevent fear that was successfully extinguished from returning post treatment [90, 91].

Increasing positive affect in chronic pain patients thus may have beneficial effects through limiting excessive spreading of pain-related fear and avoidance as well as through the optimization of (inhibitory) learning processes during treatment.

## Clinical Implications

In recent decades, efforts have been devoted to enhance positive affect in order to bolster its protective function. Meta-analyses demonstrated the overall effectiveness of a variety of positive psychology interventions on general well-being in healthy populations [53, 92]. The bulk of literature clearly suggests that many of these interventions are successful in inducing positive affect in individuals with pain complaints resulting in pain reduction or improved well-being despite pain [e.g., [18•, 19]. PPIs can be used effectively as standalone exercises [93–95] or combined in a positive psychology package [54]. Interestingly, administration of PPIs via online platforms, text message, or VR also generates promising results [18•, 19, 54]. Methods of administration of PPIs seem limitless, and they offer the possibility to provide a low-cost internet-based treatment with or without guidance of a therapist [18•, 19, 54].

Positive interventions have been found to be a worthy alternative for the gold standard in pain treatment, cognitive-behavior therapy [18•]. However, since positive and negative affect may have unique roles in modulating the experience of pain [16••, 29, 30], a treatment combining insights from both research areas may have additional benefits. This combination can be found in recent acceptance-based cognitive-behavioral treatments, in which both cognitive-behavioral techniques and elements from positive psychology are included. Preliminary evidence in depressive individuals indicates that positive affect might be one of the mediating processes leading to greater well-being and less depressive feelings following such an acceptance-based cognitive-behavioral treatment [96]. Veehof and colleagues [97] concluded that acceptance-based cognitive-behavioral treatments and traditional cognitive-behavioral treatments show comparable results in the treatment of chronic pain. Research should therefore aim at further exploring the differential effects of positive and negative emotions in order to maximize existing pain treatments.

Although cognitive-behavioral exposure treatments have proven successful, not all patients benefit from it equally and clinical practice definitely faces certain challenges. For instance, it seems the treatment may be too demanding/unpleasant for some patients. Additionally, fear is not reduced sufficiently in all patients by the end of the treatment or returns after a successful therapeutic trajectory [7–9]. Positive affect may help to tackle some of the challenges faced in the treatment of pain patients. First and most importantly, positive affect may enhance learning processes, leading to faster and more profound extinction of pain-related fear [87••]. Second, positive affect might fuel positive cognitions such as positive expectations regarding treatment outcomes. The impact of patients’ expectations for recovery on actual treatment success has repeatedly received support [98]. Following predictions from the expectancy-value model of motivation, confidence regarding the attainability of a desired goal (e.g., treatment goal) fosters action and continued effort towards this goal [99]. Third, positive affect may therefore also enhance motivation and treatment adherence, which are important predictors of the success of exposure treatments [100]. In clinical practice, it is well known that motivating patients to expose themselves to feared movements (that initially might even increase pain) during the sessions and in everyday life is challenging yet necessary.

Thus, the use of PPIs in clinical practice definitely offers a myriad of possibilities. PPIs are effective and could easily be introduced in any treatment to enhance treatment effects or to prevent dropout (by means of shaping participants’ motivation or treatment expectations). Moreover, access to positive psychology prior to, during, or post intervention might offer solutions to problems such as long waiting lists, passive treatment participation, or difficulties to maintain long-term treatment effects.

## Conclusion

Only recently, attention has grown for the role of protective factors in (pain) psychology [14, 15••]. Evidence has accumulated that not only negative affect but also positive affect might play a substantial role in determining pain patients’ well-being [16••, 32]. Evidence supporting positive affect as a resiliency factor for chronic pain is expanding. Not only does positive affect lead to lower pain intensity ratings and higher pain tolerance [32, 52], it also diminishes pain disability and improves quality of life [31, 42, 46]. Interventions enhancing positive affect have been shown to reduce pain and improve the well-being of pain patients [e.g., 18•, 19]. Moreover, it has been suggested that enhancing positive affect may have the potential to optimize treatment efficacy of existing pain treatments [87••]. Comprehensive understanding of both risk and resilience factors for pain on processes involved in pain and in the treatment of pain might lead to most effective combined strategies in clinical practice.
